# Texas Asylums in Politics

**Published:** 1895-03

**Authors:** James Orr


### TEXAS MEDICAL JOURNAL.

ESTABLISHED JULY, 1885.

PUBLISHED MONTHLY.—SUBSCRIPTION $2.00 A YEAR.

VOL. X. AUSTIN, MARCH, 1895. NO. 9.

### Original Contributions.

For Texas Medical Journal.

### TEXAS ASYLUMS IN POLITICS.

BY JAMES ORR, M. D.

Read before the Terrell Medical Society.

MR. PRESIDENT:—I desire to call the attention of this society and through them that of the people generally, to what seems to me an imperative demand for radical reformation in prevalent methods of conducting our asylums for the insane, that most unfortunate of all classes of the afflicted, who with reason dethroned and mental co-ordination gone, stand as jabbering idiots, helpless and often hopeless cares upon the bounty of loving relatives or the Christian charity of a civilized people.

In offering this paper I desire to say at the outset, that I do so without in any manner intending a reflection of any kind whatever upon these physicians who have had sufficient "pull" to enable them to be recipients of political preferment at the hands of the lately inaugurated administration. I am not writing for, or against anybody, but in the interest of medical progress, in the cause of humanity, and against a practice that is wrong. In this light I desire this paper considered.

The first point I wish to mention is the insufficient medical attention allowed these poor people, and I know of no better way of doing so than by using the asylum at this place as an illustration. Here we have over 900 people, 800 of whom, sick in both body and mind, have been sent here for medical care, and only three physicians to treat them. Of these the superintendent has so much of his time occupied with the numerous details of governing and controlling the institution that he can do little more than act as consultant and advisor of his assistants, consequently we find the medical attention of this large number of insane patients devolving on two assistants who are often inexperienced practitioners. It requires a very brief calculation to show you that one man can not under any circumstances visit and do justice to 400, or the half of that many patients per day; as a result only those in bed sick, or so violent or unruly that the attendants can not handle them, ever receive any professional treatment. It is no fault of medical attendants that they do not receive it, but the fault of the politicians running the State, who are always ready to make political capital by applying the pruning knife of economy to every appropriation asked for for those poor non-voting and uninfluential unfortunates.

It is no use to say that all do not need medical attention, for they do. When any person is crazy he needs it, and if better professional care were given these people the percentage of cures would be greater. This alone should be sufficient incentive to the adoption of a more liberal course in this matter, yet it is but a little in comparison with the lasting good to be obtained in this vast field for medical research and investigation, which is now practically neglected, because those in charge have no time to study cases carefully, to accurately arrange statistics or comparisons or communicate their findings to the medical profession.

Mr. President, I do not wish to be considered extravagant in my calculations, but I honestly believe that an asylum as large as this one, should have at least six sub-assistants added to its present staff, in order to secure anything like adequate medical attention to those entrusted to it, in addition to which the unlimited field for research and investigation would enable them to collect and disseminate information nowhere else attainable, that would be of untold benefit to this, as well as future generations. Less than $10,000 per annum would pay for this additional medical service, the benefits of which could not be measured by ten times that amount.

The next thing to which I wish to direct your attention is the necessity for removing the control of institutions from the domain of partisan politics. "To the victors belong the spoils," should not apply here, where of all other places experience is of so much importance. No one should be placed upon the staff of any asylum who does not expect to make neurology a specialty, and the physician who takes one of these places "just for the pay there is in it," as a reward for political support, or for the sake of holding an office, does an injustice to himself, to the profession, to those in his care, and to mankind generally.

A few years ago the legislature very wisely sought to remove the asylums from politics, by providing for the appointment of boards of managers for them who were to serve six years, so arranged, however, that a majority of the old board should always hold over; to these boards were given the entire management and control of the asylums. Under this law they are required to elect the the superintendent "who shall be a physician *skilled in the treatment of the insane*." The superintendent nominates his assistants, etc., who are subject to confirmation by the board. Governor Ireland approved this law, and faithfully carried out its provisions, but Governor Ross, finding it in his way, declared that feature allowing boards to hold over unconstitutional, that he might get a board to carry out his wishes in charge of the Austin asylum. The Terrell asylum was not interfered with, and the old board continued its management until Governor Hogg assumed the reins of State, when, *presto*. He selected all leading employes, and then appointed boards to whom his word was law, and no one could be appointed or removed without his consent; thus were the wheels turned backward, and when Mr. Culberson was nominated, he pledged himself to carry out the policies of his predecessor, which he has faithfully done in the management of the asylums, for he gave out the names of his appointments even to assistant places in the various asylums several weeks before his inauguration. Of course every one who has read the law knows that the governor has no right or the authority to name the superintendent at Austin and Terrell, and that his assumption of the power to name assistant superintendents, stewards, matrons, etc., is, if possible, a greater violation of a plain statute. I sometimes find myself asking the question if any governor would have thus trampled the rights of lawyers under foot.

These actions are one of the demands of the spoils system, and let us see how it works. Dr. Preston, with his experience and eminently successful management, is removed, to make room for a personal and political friend of the governor, who is wholly and entirely *without asylum experience.* Dr. F. S. White, who has had eleven consecutive years of experience in asylums in Texas, and whose annual report makes the best showing in the State, is made to stand aside for one who has no experience whatever in the treatment of the insane or managing any large institution.

I have nothing to say against these gentlemen who are so fortunate, but I condemn the system that permits such use of the greatest eleemosynary institutions of the State, and I know of no better way of remedying this matter than by a change in the organic law of the State placing all the penal and charitable institutions under the supervision of a Board of Charities and Corrections, elected by the people, as the Railroad Commissioners are to be, so a majority will hold over at each election. Such a board could manage all the State asylums and prisons much more cheaply, efficiently and systematically than now. Of course, such a change would deprive the governor of a lot of patronage, but no public service was ever injured by such deprivation.

The next thing to which I wish to direct attention, is, while asylums are built and supported for the care of the sick and afflicted, no physician has, except Dr. Graves, at San Antonio, ever been appointed on the board of managers; yet who is better qualified to judge of the fitness of men or measures in such places than a physician? The reason for discriminating against doctors on these boards is two-fold: First, there is a little rivalry or jealousy between the professions of law and medicine; the lawyer governors are inclined to tardiness in recognizing us as parts of their administration, or necessary parts of any political system. Secondly, doctors cannot be depended upon to carry out the policies of governors in running these institutions when they are different from their own conceptions of right and necessity; hence, doctors are not wanted on these boards, notwithstanding they are medical establishments.

Lastly, I wish to mention the policy governing appropriations for these asylums, which is aptly illustrated in the actions of the last governor, who approved bills establishing costly and sometimes useless courts, costly commissions and superfluous boards, but vetoed the appropriation, on the ground of economy, for a hospital for sick and dying lunatics, or by Governor Culberson's message, in which his largest percentage of reductions in State expenses is made in the items of asylums.

To recapitulate, I believe the cause of humanity, the interests of science and the good of the whole people would result in taking our asylums out of partisan politics, electing the managers, one of whom shall be a physician as above indicated; increase the medical attendance sufficiently to allow every inmate to receive constant supervision and careful attention; to make sufficient appropriation to generously provide for the care and maintenance of these poor people; to collect for the benefit of mankind all the information obtainable by a careful study of their unfortunate conditions. The attainment of these ends should enlist the co-operation of every lover of medical science, every believer in Christian philanthropy, indeed, of every patriotic citizen regardless of personal or political preferences.

